# BMSC-derived extracellular vesicles intervened the pathogenic changes of scleroderma in mice through miRNAs

**DOI:** 10.1186/s13287-021-02400-y

**Published:** 2021-06-05

**Authors:** Jiahui Jin, Qingjian Ou, Zhe Wang, Haibin Tian, Jing-Ying Xu, Furong Gao, Shuqin Hu, Jie Chen, Juan Wang, Jieping Zhang, Lixia Lu, Caixia Jin, Guo-Tong Xu, Jingjun Zhao

**Affiliations:** 1 Department of Dermatology, Tongji Hospital, School of Medicine, Tongji University, Shanghai, 200065 China; 2 Department of Ophthalmology of Shanghai Tenth People’s Hospital, and Laboratory of Clinical Visual Science of Tongji Eye Institute, School of Medicine, Tongji University, Shanghai, 200072 China; 3 Translational Medical Center for Stem Cell Therapy and Institute for Regenerative Medicine, Shanghai East Hospital, School of Life Sciences and Technology, Tongji University, Shanghai, 200120 China

**Keywords:** Scleroderma, Fibrosis, Bone marrow mesenchymal stem cell, Extracellular vesicles

## Abstract

**Background:**

Systemic sclerosis (SSc) is a disease that features severe fibrosis of the skin and lacks effective therapy. Bone marrow mesenchymal stem cell (BMSC)-derived extracellular vesicles (EVs) are potential stem cell-based tools for the treatment of SSc.

**Methods:**

BMSCs were isolated from the bone marrow of mice and identified with surface markers according to multilineage differentiation. EVs were isolated from the BMSC culture medium by ultracentrifugation and identified with a Nanosight NS300 particle size analyzer, transmission electron microscopy (TEM), and western blot. The microRNAs (miRNAs) of BMSC-derived EVs (BMSC-EVs) were studied via miRNA sequencing (miRNA-seq) and bioinformatic analysis. An SSc mouse model was established via subcutaneous bleomycin (BLM) injection, and the mice were treated with BMSCs or BMSC-derived EVs. Skin tissues were dissociated and analyzed with H&E staining, RNA sequencing (RNA-seq), western blot, and immunohistochemical staining.

**Results:**

Evident pathological changes, like fibrosis and inflammation, were induced in the skin of BLM-treated mice. BMSCs and BMSC-EVs effectively intervened such pathological manifestations and disease processes in a very similar way. The effects of the BMSC-EVs were found to be caused by the miRNAs they carried, which were proven to be involved in regulating the proliferation and differentiation of multiple cell types and in multiple EV-related biological processes. Furthermore, TGF-β1-positive cells and α-SMA-positive myofibroblasts were significantly increased in the scleroderma skin of BLM-treated mice but evidently reduced in the scleroderma skin of the EV-treated SSc group. In addition, the numbers of mast cells and infiltrating macrophages and lymphocytes were evidently increased in the skin of BLM-treated mice but significantly reduced by EV treatment. In line with these observations, there were significantly higher mRNA levels of the inflammatory cytokines Il6, Il10, and Tnf-α in SSc mice than in control mice, but the levels decreased following EV treatment. Through bioinformatics analysis, the TGFβ and WNT signaling pathways were revealed to be closely involved in the pathogenic changes seen in mouse SSc, and these pathways could be therapeutic targets for treating the disease.

**Conclusions:**

BMSC-derived EVs could be developed as a potential therapy for treating skin dysfunction in SSc, especially considering that they show similar efficacy to BMSCs but have fewer developmental regulatory requirements than cell therapy. The effects of EVs are generated by the miRNAs they carry, which alleviate SSc pathogenic changes by regulating the WNT and TGFβ signaling pathways.

**Supplementary Information:**

The online version contains supplementary material available at 10.1186/s13287-021-02400-y.

## Introduction

Scleroderma (also known as systemic sclerosis, SSc) is an autoimmune connective tissue disease with unknown etiology and is characterized by three hallmark characteristics (vasculopathy, immune dysfunction, and fibroblast dysfunction) that which results in excessive accumulation of collagen and fibrosis in the skin and visceral organs [1, 2]. Since there is no effective therapy, scleroderma often causes severe disability and even death [3, 4]. Therefore, in-depth studies on the etiology of and effective treatments for scleroderma are necessary to improve the quality of life and life expectancy of patients.

Inflammation and vascular injury are reported to drive the autoimmune response and precede fibrosis in the initial stages of SSc [5, 6]. This fibrosis is regulated by a combination of autocrine and paracrine profibrotic mediators, such as transforming growth factor-β1 (TGF-β1), interleukin 4 (IL-4), interleukin 13 (IL-13), and interleukin 10 (IL-10). These mediators secreted by macrophages and monocytes promote tissue-resident fibroblasts to differentiate into myofibroblasts and enhance the production of collagen and other extracellular matrix (ECM) components by local fibroblasts and α-smooth muscle actin (α-SMA)-positive myofibroblasts in the affected organs [6–8]. Although SSc is considered an autoimmunity disease, toxin exposure and viral infection can also induce its occurrence and development. Subcutaneous injection of bleomycin (BLM) can induce skin fibrosis in mice that is similar to that seen in human SSc, including dermal fibrosis and abnormalities in ECM deposition, and this model is the most widely used preclinical animal model in antifibrotic research [9, 10].

Many regenerative medical therapies, such as mesenchymal stem cell (MSC) transplantation, have been explored for relieving the symptoms of or curing this difficult disease [11]. Alexandre et al. reported that transplanted allogeneic or xenogeneic bone marrow MSCs (BMSCs) demonstrated similar antifibrotic therapeutic effects in SSc [12]. Our previous report also showed that transplanted BMSCs or genetically engineered BMSCs attenuated skin fibrosis and reactive oxygen species (ROS)-induced apoptosis in the BLM-induced murine SSc model [10]. However, the number of colony-forming units in and the differentiation efficiency of the MSCs in the SSc injury site were relatively low. MSCs may exert their effects not through their differentiation but through paracrine mechanisms, such as extracellular vesicles (EVs), which contain cytokines, signaling lipids, and regulatory microRNAs (miRNAs) involved in cellular communication [13–15]. Recently, EVs, which are released by the outward budding of various types of cells (including MSCs), have been identified to contain major paracrine factors and thus could be appealing candidates as vectors for cell therapy [13, 14]. Intracellular delivery of EVs has been demonstrated for a number of different cell types and allows the functional utilization of the delivered miRNAs [16]. EVs have been implicated in the regenerative effects of MSCs in a wide variety of tissues, including skin, muscle, lung and vascular tissues [17–20]. MSC-derived EVs have also shown therapeutic potential in fibrotic diseases, such as renal fibrosis, corneal fibrosis, myocardial fibrosis, and hepatic fibrosis [21–23]. However, it is still unclear whether BMSC-derived EVs can mediate skin fibrosis in SSc.

In the present study, as a continuation of previous studies, we investigated the effects of subcutaneous injection of BMSC-derived EVs as a treatment for BLM-induced SSc mice and the underlying mechanism. BMSC-derived EVs significantly relieved fibrosis and inflammation in the skin, similar to the effects of BMSC transplantation. We also identified a group of specific miRNAs in BMSC-derived EVs. Multidimensional bioinformatics analysis suggested that these miRNAs contribute to the inhibition of α-SMA expression and collagen deposition, as well as fibroblast/myofibroblast transition-induced fibrosis and inflammation. Therefore, BMSC-derived EVs could be a potential therapeutic strategy for alleviating inflammation and skin fibrosis in patients with SSc.

## Methods

### Experimental animals

All procedures using animal subjects were performed in accordance with the Guide for the Care and Use of Laboratory Animals, and the experiments were approved and performed following the guidelines of the Institute of Laboratory Animal Resources, Tongji University. Female C57BL/6 mice were obtained from Shanghai SLAC Laboratory Animal Co., Ltd. (China) and used in this study. Skin samples for molecular and histologic analyses were obtained at the time of euthanasia.

### BMSC isolation and culture

BMSCs were isolated from the bone marrow of the femurs and tibias of mice (4 weeks old). The BMSCs were cultured in α-MEM medium (SH30265.01, HyClone, Thermo Fisher Scientific, USA) supplemented with 15% fetal bovine serum (FBS; 10091148, Thermo Fisher Scientific, USA) and 100 U/mL penicillin-streptomycin solution (10378016, Thermo Fisher Scientific, USA). The BMSCs were passaged when they reached 80% confluence. BMSCs in passage 3 were used in the analysis and for the production of EVs.

### Flow cytometry analysis

BMSCs in passage 3 were used for flow cytometry analysis. Flow cytometry analysis of BMSC surface markers was performed as follows. BMSCs were suspended in phosphate-buffered saline (PBS; E607008-0500, Sangon Biotech, China) at a final concentration of 1 × 10^6^/mL. Then, monoclonal antibodies and the isotype control were added to 100 μL cell suspensions and incubated for 60 min at 4 °C. The cell suspensions were centrifuged at 2000 rpm for 3 min to remove the antibodies and washed with PBS 3 times. Finally, 500 μL PBS was used to resuspend the cell pellet, which was analyzed with the CytoFLEX LX system (Beckman Coulter, USA). FlowJo software (Leonard Herzenberg Laboratory, USA) was used to analyze flow cytometry data.

### Multilineage differentiation of BMSCs

Mouse BMSCs in passage 3 were cultured in differentiation conditions to identify their capacity for multilineage differentiation. To induce adipogenic differentiation of BMSCs, when the cells reached 70 to 80% confluency, the complete medium was replaced with adipogenic induction medium (DMEM supplemented with 10% FBS, 10^−7^ M dexamethasone (D1756, Sigma Aldrich, Germany), 10 mM β-glycerol phosphate (G9422, Sigma Aldrich, Germany), 50 μM L-ascorbic acid 2-phosphate (49752, Sigma Aldrich, Germany), and 10 μg/mL insulin (I0320000, Sigma Aldrich, Germany)), and the cells were cultured for 3 weeks. Then, Oil Red O staining was performed.

To induce osteoblastic differentiation of BMSCs, when the cells reached 60 to 70% confluency, the complete medium was replaced with osteogenic induction medium (DMEM medium (SH300022.01, HyClone, USA) supplemented with 10% FBS, 10 mM β-glycerol phosphate (G9422, Sigma Aldrich, Germany), 50 μM L-ascorbic acid 2-phosphate (49752, Sigma Aldrich, Germany), 100 ng/mL recombinant human bone morphogenic protein-2 (120-02, Peprotech, USA), and 10^−7^ M dexamethasone (D1756, Sigma Aldrich, Germany)), and the cells were maintained in this medium for the next 21 days. Then, alizarin red staining was performed.

To induce chondrogenic differentiation of BMSCs, when the cells reached 70% confluency, the complete medium was replaced with chondrogenic induction medium (DMEM supplemented with 10% FBS, 50 μg/mL L-ascorbic acid 2-phosphate (49752, Sigma Aldrich, Germany), 100 μg/mL sodium pyruvate (P5280, Sigma Aldrich, Germany), 40 μg/mL proline (P3350000, Sigma Aldrich, Germany), 10 ng/mL TGF-β1 (100-21, Peprotech, USA), 10^−7^ M dexamethasone (D1756, Sigma Aldrich, Germany), and 100 ng/mL insulin-like growth factor-1 (IGF-1, 100-11, Peprotech, USA)), and the cells were cultured for 3 weeks. Then, toluidine blue staining was performed.

### Isolation and identification of BMSC-derived EVs

The mouse BMSCs in passage 3 were cultured in α-MEM (SH30265.01, HyClone, USA) supplemented with EV-depleted FBS and 100 U/mL penicillin-streptomycin solution (10378016, Thermo Fisher Scientific, USA). EV-depleted FBS was depleted of EVs by ultracentrifugation for 14 h at 150,000*g*. Then, the supernatant was separated and used as EV-depleted FBS. The BMSC culture medium was collected every 48 h. The collected culture medium was centrifuged at 300*g* for 10 min at 4 °C to eliminate cell pellets. The supernatant was centrifuged at 2000*g* for 20 min at 4 °C to further remove cell debris. Then, the supernatant was again centrifuged at 10,000*g* for 30 min at 4 °C. The supernatant was then filtered through a 0.22-μm filter (GSWP04700, Merck, Germany), and the flow through was transferred to new tubes and ultracentrifuged at 150,000*g* for 2 h at 4 °C in a SW70Ti rotor (Beckman Coulter, USA) to pellet the EVs. The supernatant was immediately aspirated upon completion of the first ultracentrifugation and then ultracentrifuged again as described previously. For maximal EV retrieval, the EV-enriched pellet was resuspended in 200 μL cold PBS. The concentration of EVs was measured according to the protein content using a BCA protein assay kit (23227, Thermo Fisher Scientific, USA). The presence of EVs was confirmed with a NanoSight NS300 instrument (Malvern Instruments, UK). Transmission electron microscopy (TEM; Tecnai 12, FEI, USA) and western blot were employed to detect morphology and surface markers.

### Establishment and treatment of the SSc model

BLM (R25001, Thermo Fisher Scientific, USA) was diluted in PBS at a concentration of 1 mg/mL and sterilized by filtration. To establish the murine fibrosis model, 100 μL BLM solution was subcutaneously injected into the shaved backs (1 cm^2^) of mice using a 27-gauge needle. Injections were made once a day for 28 consecutive days. Mice in the control group received 100 μL PBS.

For the BMSC or BMSC-EV treatments, BLM-induced SSc mice were randomly divided into 3 groups (6 mice in each group) and treated with PBS (100 μL), BMSCs (1 × 10^6^/100 μL), or BMSC-derived EVs (15 μg/100 μL). The numbers of BMSCs used in this study were determined according to our previous report [10]. BMSC-EVs (15 μg) were produced from 2 × 10^6^ BMSCs to ensure a treatment effect. The mice were killed 14 days after treatment, and skin tissue samples were collected from a 1 cm^2^ shaved area.

### Fibroblast cell culture and treatment

Mouse fibroblast cells were isolated from mouse skin with 0.25% trypsin and cultured in DMEM/F12 (D8437, Sigma Aldrich, Germany) with 10% FBS (10091148, Thermo Fisher Scientific, USA). Fibroblasts were passaged and treated with 10 ng/mL TGF-β1 or a combination of TGF-β1 (10 ng/mL) and BMSC-EVs (50 μg/ml). After that, the cells were lysed with RIPA buffer (P0013B, Beyotime, China) containing protease and phosphatase inhibitor cocktails (C0001 and C0004, TargetMol, USA) and collected for western blot analysis.

### Histochemical analysis

Skin tissue samples from different groups were fixed in 4% paraformaldehyde (PFA; E672002-0500, Sangon Biotech, China) solution for 24 h. Then, the tissues were embedded in paraffin and cut into 10-μm sections. The sections of the paraffin-embedded skin tissue were deparaffinized using xylene and rehydrated using decreasing concentrations of ethanol (100, 95, 85, and 75%). Briefly, the sections were stained in hematoxylin for 5 min and further washed with cold running water. After incubation with 1% hydrochloric acid-alcohol, the sections were washed and stained with 0.5% eosin dye solution. To analyze the extent of skin fibrosis, randomly selected fields of the sections were captured. The sections were stained with Masson trichrome staining (BP-DL023, SenBeiJia Biological Technology Co., Ltd, China) according to the manufacturer’s instructions, and the collagen fibers were evaluated under a light microscope. For detection of mast cells, the sections were stained with toluidine blue. Sections were examined and photographed using a microscope (TI2-E, Nikon, Japan).

For the inflammatory cell counts, the hematoxylin-stained skin sections were examined with a light microscope at × 200 magnification. We selected 3 views of each stained skin section randomly and quantified the positive stained cells by carefully counting in 0.5 mm broad, band-like area below and parallel with the dermal–epidermal junction.

### Immunohistochemical analysis

The sections of paraffin-embedded skin tissue were deparaffinized as previously described [24]. The sections were incubated in 3% H_2_O_2_ for 5 min at room temperature. Then, 5% goat serum (E510009, Sangon Biotech, China) was used to block the sections for 60 min at room temperature. The primary antibodies listed in Supplemental Table 1 were diluted in 5% goat serum solution, added to the sections, and incubated overnight at 4 °C. Horseradish peroxidase (HRP)-labeled secondary antibodies were added and incubated for 60 min at room temperature. Diaminobenzidine (DAB) solution was utilized to show positive signaling.

### Hydroxyproline measurement

The collagen content of skin samples was quantified with a hydroxyproline test kit (A030-2-1, Nanjing JianCheng Bioengineering Institute, China) according to the recommendations of the manufacturer. Hydroxyproline content was determined with the following formula: (tested OD value - blank OD value) / (standard OD value − blank OD value) × standard sample concentration (5 μg/mL) × total hydrolysate volume (10 mL) / tissue wet weight (mg).

### Western blotting analysis

Samples were lysed with RIPA buffer (P0013B, Beyotime, China) supplemented with protease and phosphatase inhibitor cocktails (C0001 and C0004, TargetMol, USA). Total protein (20 μg) was separated by SDS-PAGE (10%) and transferred to PVDF membranes (IPFL85R, Merck, Germany). After being blocked in 5% nonfat milk for 1 h, the membranes were incubated with primary antibodies (as listed in Supplemental Table 1) at 4 °C overnight. After being washed, the membranes were incubated with HRP-conjugated secondary antibodies for 1 h at room temperature. Signals were detected using a Tanon chemiluminescence image detection system (5200S, Tanon, China).

### Quantitative real-time PCR

Total RNA was extracted and purified using TRIzol reagent (9109, Takara, Japan) according to the manufacturer’s instructions, and cDNA was synthesized using PrimeScript RT Master Mix (RR036A, Takara, Japan) according to the manufacturer’s instructions. Quantitative real-time PCR was performed using SYBR Green Real-time PCR Master Mix (FP205-03, Tiangen, China). The oligonucleotide primers (Sangon Biotech, China) that were used are listed in Supplemental Table 2. Gene expression was normalized to the average value of GAPDH, β-ACTIN, and 18S mRNA in each sample. The fold change in expression was calculated using the 2^ΔΔCt^ method.

### RNA sequencing (RNA-seq) and bioinformatic analysis

For miRNA sequencing (miRNA-seq) of EVs, preparation of tagged miRNA-seq libraries, sequencing, and next-generation sequencing (NGS) data analysis were performed by LC Sciences (USA). The library was sequenced with the Illumina Hiseq 2500 SE50 platform. Raw reads were subjected to an in-house program, ACGT101-miR (LC Sciences, USA), to remove adapter dimers, junk, low-complexity, and common RNA families (ribosomal RNAs, transfer RNAs, small nuclear RNAs, and small nucleolar RNAs) and repeats. Subsequently, unique sequences with lengths of 18~26 nucleotides were mapped to specific species precursors in miRBase 22.0 via a BLAST search to identify known miRNAs and novel 3p- and 5p-derived miRNAs. A criterion that the number of reads was higher than the average copy number of the dataset was used to filter the high-level miRNAs. The R package multiMiR (version 3.12) was used for miRNA target scanning and prediction [25], while clustering analysis of the target genes was performed using the R package clusterProfiler.

For RNA-seq of tissue samples, skin tissue was dissected under a microscope and immediately placed in TRIzol reagent. Total RNA was isolated with TRIzol reagent (9109, Takara, Japan). The library was sequenced with an Illumina NovaSeq 6000 PE150. The criteria |logFC| > 1 and P < 0.05 were applied to filter the differentially expressed genes. Gene ontology (GO) and Kyoto Encyclopedia of Genes and Genomes (KEGG) analyses were performed with the R package clusterProfiler. The results were visualized with GOplot.

### Statistical analysis

All data are expressed as the standard error of the mean. Data analysis was performed using GraphPad Prism software (USA). One-way ANOVA was employed for the statistical comparison. A value of *P* < 0.05 was considered statistically significant. In the figures, asterisks are used to express the statistical significance of values: *: *P* < 0.05, **: *P* < 0.01 and ***: *P* < 0.001. In the results, detailed P values are provided unless *P* < 0.001.

## Results

### Identification of mouse BMSCs and BMSC-derived EVs

**Fig. 1:**
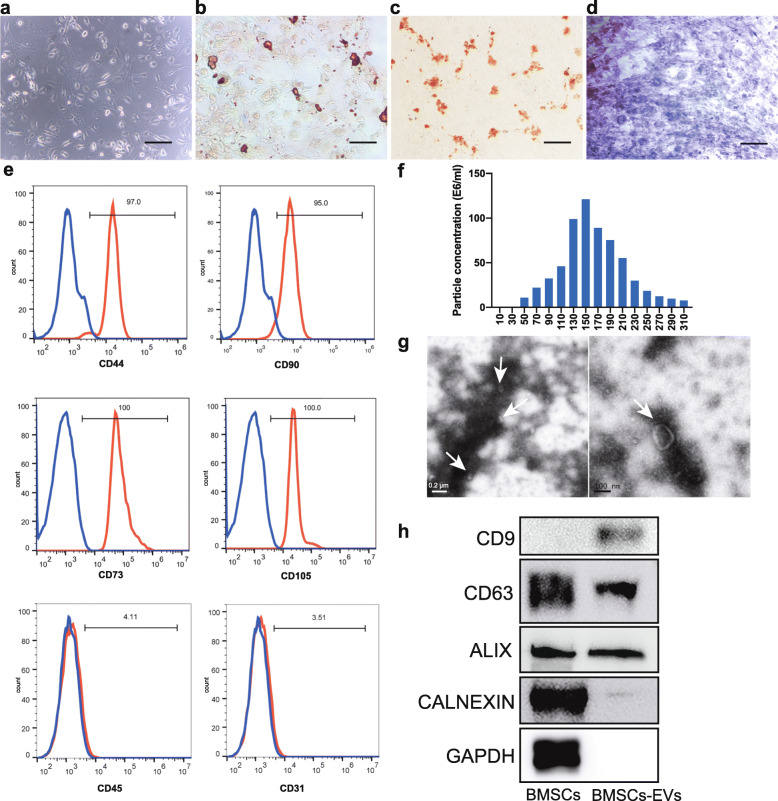
Identification of mouse BMSCs and BMSC-derived EVs. **a** Morphology of BMSCs cultured in regular medium at passage 3. Scale bar 100 μm. **b** Oil Red O staining of BMSCs induced to undergo adipogenic differentiation for 21 days. Scale bar 200 μm. **c** Alizarin red staining of BMSCs induced to undergo osteogenic differentiation for 21 days. Scale bar 200 μm. **d** Toluidine blue staining of BMSCs induced to undergo chondrogenic induction for 21 days. Scale bar 200 μm. **e** Flow cytometry plots showing that the BMSCs were positive for CD44, CD105, CD90, and CD73, and negative for CD31, CD45. **f** Particle size distribution of BMSC-derived EVs measured by nanoparticle tracking analysis (NTA). **g** Morphology of EVs on transmission electron microscopy (TEM). **h** Western blot analysis of the protein profiles of EVs. BMSCs: bone marrow mesenchymal stem cells; EVs: extracellular vesicles derived from mouse BMSCs

Following isolation and culture as described above, mouse BMSCs were identified according to the criteria of the International Society of Cellular Therapy [26]. As shown in Fig. 1a, the isolated mouse BMSCs adhered to the culture dish with fibroblast-like and spindle-shaped morphology. After adipogenic induction, small cytoplasmic lipid droplets in BMSCs were observed upon Oil Red O staining (Fig. 1b). After osteoblast induction, positive alizarin red staining could be seen (Fig. 1c). After chondrogenic induction, toluidine blue staining was observed (Fig. 1d). The surface markers of the BMSCs were analyzed with flow cytometry, and the results are shown in Fig. 1e. BMSCs highly expressed CD73 (100%), CD105 (100%), CD44 (97%), and CD90 (95%), while CD31 and CD45 were negatively expressed. Thus, the mouse BMSCs exhibited typical mesenchymal stem cell characteristics.

The EVs isolated from the mouse BMSC culture media were analyzed with a NanoSight NS300 particle size analyzer. As shown in Fig. 1f, most of these EVs ranged from 90 to 230 nm in size, and they appeared as round or elliptical vesicles of uneven size with intact capsules under TEM (Fig. 1g). Moreover, the EV-specific markers CD9, CD63, and ALIX were significantly enriched in these EVs. CALNEXIN and GAPDH were not expressed by the EVs (Fig. 1h). Taken together, these data show that mouse BMSCs and BMSC-derived EVs were isolated successfully.

### Administration of BMSCs or BMSC-EVs reduces BLM-induced dermal thickening and fibrosis

**Fig. 2:**
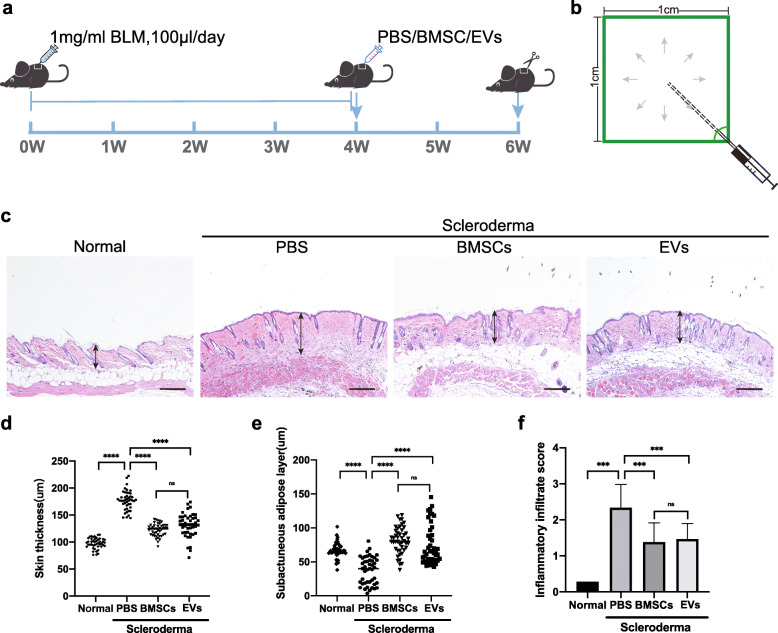
BMSC and BMSC-EV treatments reduce bleomycin-induced dermal thickening and fibrosis. **a** Schematic showing the mouse SSc model generation and treatment process. **b** Schematic showing the injection technique and strategy for distributing bleomycin, BMSCs, and BMSC-EVs as evenly as possible. **c** H&E staining of representative skin sections from mouse skin tissue. Arrows show the dermal thickness. Scale bar 200 μm. **d, e** The thicknesses of the dermis skin and subcutaneous fat layer were measured with ImageJ software in H&E-stained images. **f** Inflammatory cells were counted and identified by H&E staining. SSc: Systemic sclerosis

To confirm the effects of BMSCs in SSc that we previously reported and examine whether mouse BMSC-EVs have any effects on BLM-induced SSc pathology, a mouse model was established according to the following protocol: daily subcutaneous injection of BLM for 4 weeks, followed by subcutaneous injections of mouse BMSCs or BMSC-EVs (Fig. 2a). BLM, BMSCs, and BMSC-EVs were subcutaneously injected into the centers of shaved skin areas for even distribution of the liquid and effects (Fig. 2b).

Two weeks after the BMSC and EV treatments, in comparison with the respective layers of normal mouse skin, the dermis layer of PBS-treated model mice was thickened (*P* < 0.001), the subcutaneous adipose layer of PBS-treated model mice was lost, and the dermal architecture of PBS-treated model mice was disrupted (Fig. 2c, d). Both the BMSC- and EV-treated skin samples showed normal thickness of the subcutaneous adipose layer, and in both cases, the hypodermic adipose layer was significantly thicker than that in the PBS-treated mice (*P* < 0.001) (Fig. 2c, e). Furthermore, when inflammatory cell infiltration was examined as another parameter to evaluate the effects of the treatments in SSc, as shown in Fig. 2f, the inflammatory cell counts in skin tissue sections in both the BMSC and EV groups were significantly lower than those in SSc mice (*P* < 0.001), indicating attenuated leucocytic infiltration. These results demonstrate that subcutaneously injected BMSC-EVs can significantly improve dermal damage and abnormalities in this SSc model, and their effects were as strong as those of mouse BMSCs.

### Administration of BMSCs and BMSC-EVs reduces abnormal deposition of collagen in a mouse SSc model

**Fig. 3:**
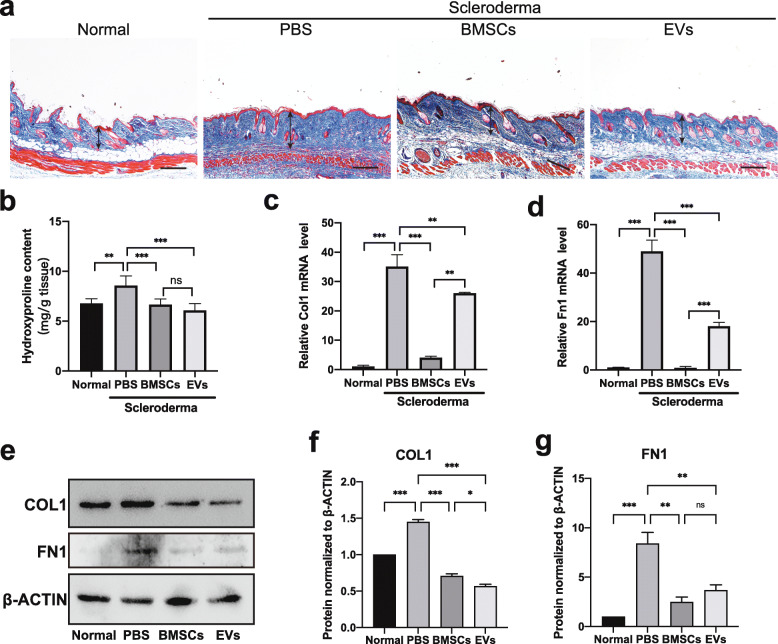
BMSC and BMSC-EV treatments reduce abnormal deposition of collagen in a mouse SSc model. **a** Masson’s trichrome staining of representative skin sections from mouse skin tissue and blue staining for collagens. Scale bar 200 μm. **b** The collagen content of skin tissues was measured by hydroxyproline assay. **c, d** mRNA levels of Col1 and Fn1 in the lesional skin were assessed by real-time PCR. **e–g** The protein expression of COL1 and FN1 in the skin was detected by western blot

Considering that abnormal ECM deposition in the dermis is an atypical characteristic of SSc, Masson’s trichrome staining was used to evaluate the ability of BMSCs and BMSC-EVs to ameliorate the deposition of excess ECM components, including collagen. As shown in Fig. 3a, BLM-induced scleroderma skin exhibited an abundance of collagen and a dense ECM structure in the dermis, but ECM deposition was significantly reduced by treatment with BMSCs or BMSC-EVs. Consistently, as shown in Fig. 3b, the BMSC (*P* < 0.001) and BMSC-EV (*P* < 0.001) treatments significantly decreased the hydroxyproline content, which was increased in the BLM-treated mice, and there was no significant difference between the BMSC group and BMSC-EV group (*P* = 0.499). Furthermore, the mRNA expression levels of Col1 and Fn1 were significantly elevated in PBS-treated SSc mice (*P* < 0.001) but were significantly reduced in mice treated with either BMSCs (*P* < 0.001) or BMSC-EVs (*P* = 0.0027) (Fig. 3c, d). The protein expression results for COL1 and FN1 were consistent with the mRNA expression results (Fig. 3e, f). However, for these two markers, the effects of BMSC-EVs were not as strong as those of BMSCs (Fig. 3c–f). These data indicate that both BMSCs and BMSC-EVs can significantly reduce BLM-induced abnormal deposition of ECM and collagen density.

### Analysis of BMSC-EVs and their specific miRNAs

**Fig. 4:**
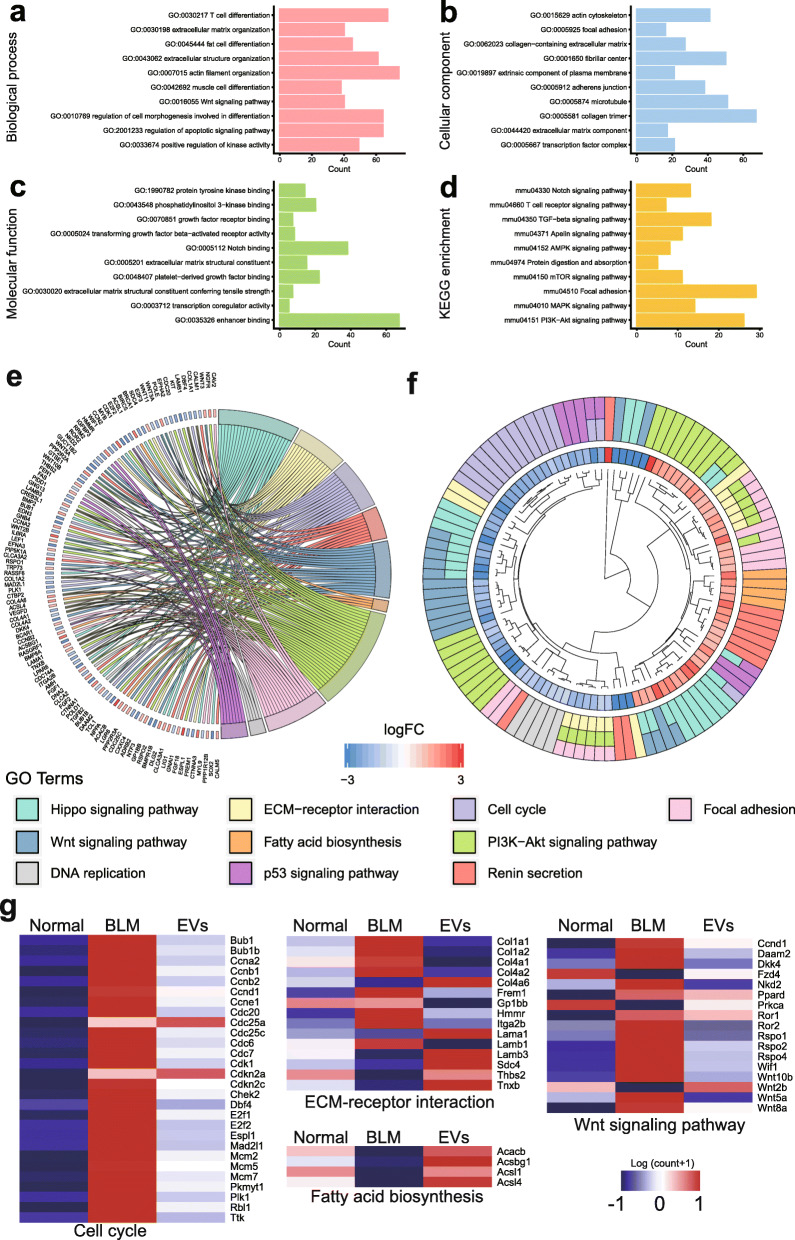
High-throughput sequencing analysis of miRNAs in BMSC-EVs and the transcriptome of skin tissues. **a–d** Gene Ontology analysis of the genes targeted by the highly expressed microRNAs in BMSC-EVs. **e,f** Gene Ontology and GO Chord analysis results showing the differentially expressed genes between BMSC-EV-treated skin and PBS-treated skin. **g** Heatmap showing the relative mRNA expression of members of related signaling pathways

Since both mouse BMSCs and their EVs attenuated the generation of disordered dermis structure and ECM deposition in the skin of BLM-induced SSc mice to a similar degree, we focused on BMSC-EVs and their functional miRNAs in the experiments below to understand the related mechanism and develop more convenient and efficient therapy. First, the global expression of miRNAs in BMSC-EVs was analyzed via high-throughput miRNA-seq approaches. As listed in Supplemental Table 3, BMSC-EVs highly expressed a cluster of specific miRNAs, such as mir-21a, mir-143, mir-27b, mir-29a, and let-7. The target genes of these highly expressed miRNAs were then studied by miRNA target scan analysis and GO functional clustering analysis to predict their potential functions. As shown in Fig. 4a, the results showed that these highly expressed miRNAs were involved in regulating the proliferation and differentiation of multiple cell types, including muscle cells, T cells, and fat cells, and in multiple processes, such as ECM generation (including processes related to collagen and cell adhesion and junctions). In addition, the WNT, TGFβ, Notch, and T cell receptor signaling pathways were also predicted to play certain roles in regulating the homeostasis of skin (Fig. 4b–d).

To confirm the functional predictions for the BMSC-EV-derived miRNAs in vivo, high-throughput RNA-seq was performed to analyze the skin tissues of BLM-treated mice with or without 7 days of BMSC-EV treatment. As shown in Supplemental Fig. 1a, in comparison with that of normal control mice, the skin of the SSc mice highly expressed genes involved in the hedgehog signaling pathway, the WNT pathway, the cell cycle and cellular senescence signaling, but the skin of BMSC-EV-treated SSc mice highly expressed genes related to focal adhesion, the Hippo signaling pathway, and fatty acid biosynthesis. Reasonably, following BMSC-EV treatment, the genes that were hyperactivated by BLM stimulation were significantly lowered, especially those in the WNT pathway, or related to ECM-receptor interactions and the cell cycle (Fig. 4e, g); however, genes related to fatty acid synthesis were upregulated. There were only a few genes that were differentially expressed between the BMSC-EV-treated group and the normal control group (Supplemental Fig. 1b).

These results suggest that the BMSC-EVs may function by regulating the WNT signaling pathway, TGFβ signaling, and inflammatory response during the treatment of BLM-induced SSc.

### BMSC-EV treatment decreases fibroblast differentiation into myofibroblasts

**Fig. 5:**
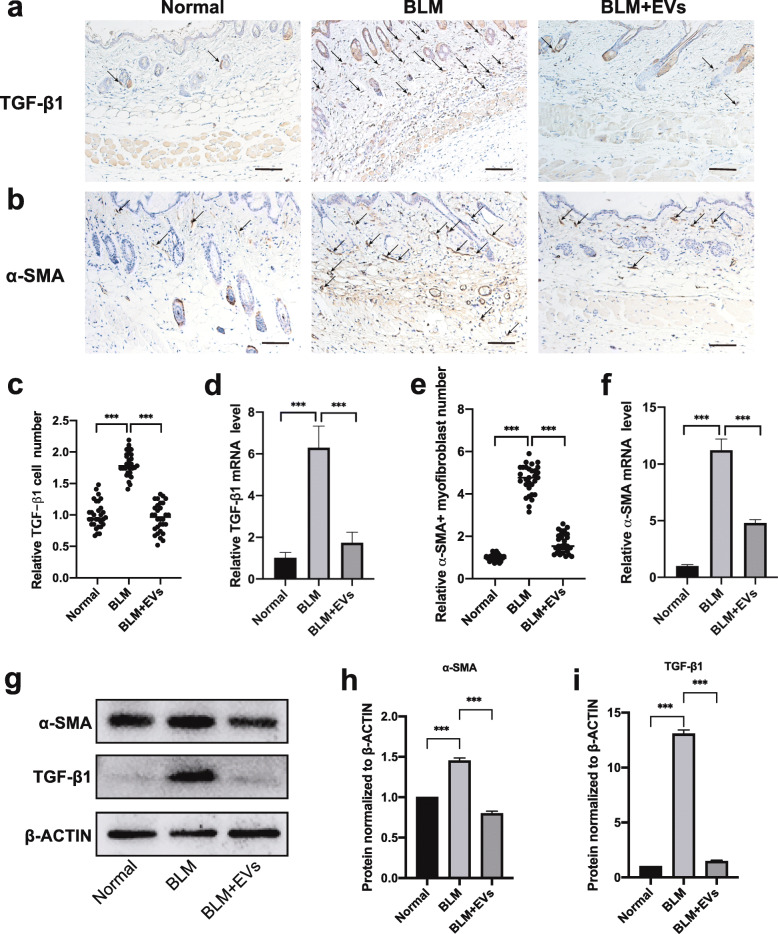
BMSC-EV treatment decreases myofibroblast differentiation into fibroblasts. **a, b** Representative images of TGF-β1- and α-SMA-positive cells detected by immunohistochemical staining. Scale bar 100 μm. **c, e** Quantitative analysis of TGF-β1- and α-SMA-positive cell numbers. **d, f** The expression levels of α-SMA and TGF-β1 were detected with real-time PCR. **g, h, i** The protein expression of TGF-β1 and α-SMA in the skin was detected by western blot. BLM: bleomycin; EVs: BMSC-derived EVs; α-SMA: α-smooth muscle actin; TGF-β1: transforming growth factor-β1

Considering the important roles of the TGFβ signaling pathway in inducing fibroblast activation and myofibroblast differentiation, both are important characteristics during the progression of BLM-induced animal models of fibrosis. As such, TGF-β1-positive cells and TGF-β1 mRNA expression were examined in the skin samples of the mice. As shown in Fig. 5a, c, there was significantly more accumulation of TGF-β1-positive cells in the dermis layer in the SSc mice than in the normal mice (*P* < 0.001), but this accumulation was evidently reduced in the EV-treated group to a level similar to that in the normal group (*P* < 0.001). The mRNA and protein levels of the groups showed similar trends (*P* < 0.001) (Fig. 5d, g).

Another typical characteristic of SSc, increased α-SMA-positive myofibroblasts in fibrotic skin [27, 28], was also examined in the same model. As shown in Fig. 5b, e, g, i, the number of α-SMA^+^ myofibroblasts was evidently increased in the scleroderma skin of BLM-treated mice (*P* < 0.001) but was reduced to normal levels in the EV-treated mice (*P* < 0.001). Consistently, the mRNA and protein levels of α-SMA in the SSc model were significantly higher than those in control mice (*P* < 0.001) and were reduced to normal levels after BMSC-EV treatment (*P* < 0.001) (Fig. 5f, g, h). These results confirm that BMSC-EVs can inhibit fibroblast activation by downregulating the expression of TGF-β1 in the dermis.

### BMSC-EV treatment reduces inflammatory infiltration in the dermis in scleroderma

**Fig. 6:**
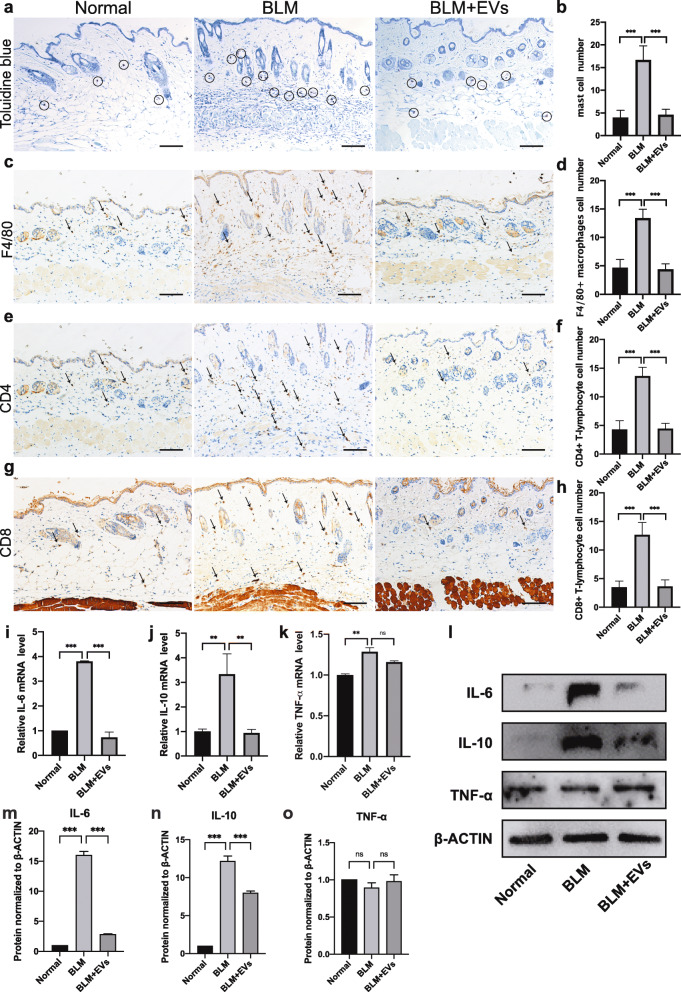
BMSC-EV treatment reduces inflammatory infiltration in the dermis in scleroderma. **a–h** Representative images of the mast cell degranulation detected by toluidine blue staining (**a, b**), F4/80^+^ positive macrophages (**c, d**), CD8^+^ and CD4^+^ positive lymphocyte (**e, f, g, h**) infiltration in the skin detected by immunostaining and their corresponding quantification. Scale bar 100 μm. **i–k** Quantification of Il6, Il10, and Tnf-α mRNA. **l–o** Protein expression of IL-6, IL-10, and TNF-α in the skin as detected by western blot. EVs: BMSC-derived EVs; BLM: bleomycin; Il6: interleukin-6; Il10: interleukin-10; Tnf-α: tumor necrosis factor α

To support our ideas above, more parameters related to SSc, like degranulation of mast cells and accumulation of macrophages and lymphocytes, were examined [29]. As shown in Fig. 6a, b, the number of mast cells in the dermis of the BLM-treated mice was significantly increased, as shown by toluidine blue staining, compared to that in normal control mice (*P* < 0.001), and BMSC-EV treatment significantly reduced the mast cell number to a level comparable to that of the normal group (*P* < 0.001). Moreover, as shown in Fig. 6c–h, immunohistochemical examination demonstrated that BMSC-EVs significantly reduced the infiltration of F4/80^+^ macrophages (*P* < 0.001) and CD4^+^/CD8^+^ lymphocytes (*P* < 0.001), and both were significantly increased in BLM-treated SSc mice.

To investigate the effects of BMSC-EVs on BLM-induced inflammation, the mRNA expression of inflammatory cytokines, including Il-10, Il-6, and Tnf-α, in skin samples was detected. The results showed that BLM-treated mice had significantly higher mRNA levels of Il-6 (*P* < 0.001), Il-10 (*P* = 0.0056), and Tnf-α (*P* = 0.0024) than normal control mice, while the levels of Il-6 (*P* < 0.001) and Il-10 (*P* = 0.0067) in the EV-treated group are significantly reduced (Fig. 6i, j). The levels of Tnf-α in the EV-treated group were not significantly different (Fig. 6k). The protein expression patterns for IL6, IL10, and TNF-α were the same as the mRNA expression patterns: the expression of IL6 and IL10 was higher in BLM-treated mice, while it was significantly reduced in the EV-treated group (Fig. 6l–o). Thus, BMSC-EV treatment can significantly reduce the infiltration of inflammatory cells and inhibit the release of inflammatory factors in the skin of BLM-induced SSc mice.

## Discussion

Curing scleroderma (SSc) is now a realistic clinical challenge [3, 4] since its etiology and pathogenesis are unclear. The key clues about its possible mechanisms are limited to vasculopathy, immune dysfunction, and fibroblast dysfunction [1, 2]. On the other hand, BMSC-based therapies, including that used in our previous work [10], have shown some therapeutic effects, like attenuating skin fibrosis and apoptosis in the BLM-induced SSc model. The observation that interested us was that the colony-forming unit number and differentiation efficiency of the BMSCs were fewer or lower in the injected sites [12]. This result may indicate that the therapeutic mechanism of these BMSCs in treating SSc involves paracrine signaling rather than differentiation. Therefore, in this study, we used BMSCs as a tool to explore the pathogenesis of scleroderma and to develop a more practical treatment by exploring BMSC-EVs and their components. Again, BLM-induced SSc mice were used as models of the disease since the pathological changes in the mice, like increased dermal thickness and collagen accumulation, are similar to those in patients with SSc [29].

MSCs have been reported to play an antifibrotic role in fibrotic diseases such as liver fibrosis [30], kidney fibrosis [31], lung fibrosis [32, 33], and skin fibrosis [10]. However, there are still unresolved and unavoidable risks of MSC clinical applications, such as iatrogenic tumor formation, cellular rejection, and infusion toxicity [34]. Additionally, the use of cells as drugs is still in a very preliminary stage, and the evaluation and approval of such drugs for clinical use are a long way off. On the other hand, EVs derived from MSCs have been shown to be key factors in MSC-to-surrounding cell communication [35] and are considered stem cell-based, cell-free drugs and carriers of siRNA [36]. Compared to BMSCs, BMSC-EVs have several advantages, like being simpler to produce and store and having easier quality control procedures. As EVs are not cells, viability is not a concern. This makes EVs potentially much easier to use than cells post thaw. Indeed, there is preliminary evidence that the thawing process may alter membranes of EVs so that they are more easily absorbed by target cells [37]. The safety of intravenous and intraperitoneal injection of EVs has been verified in animal experiments [38, 39]. However, the large-scale production of EVs is influenced by the specific therapeutic application. As products of cells, the manufacture of EVs is dependent on the ability to produce large quantities of cells in ways that do not alter certain cell behavior and characteristics. Some alterations in the cell culture platform might alter the production, composition, attributes, or function of EVs [40]. Furthermore, some primary cell lines (such as mesenchymal stem cells) exhibit a low proliferative capacity, limiting the ultimate culture size and duration, number of production batches, and reproducibility. Solutions to these issues include cell immortalization via overexpression of the MYC gene [41]. Furthermore, the safety and efficiency of MSC-derived EVs have been evaluated in several clinical trials for various diseases, including an inhaled form in severe acute respiratory syndrome coronavirus 2 (NCT04276987, ChiCTR2000030261) and an intravenous injection in diabetes mellitus (NCT02138331). Therefore, we designed the present study to determine whether BMSC-EVs can mediate the effects of BMSCs in treating SSc with the hope that they can be developed into a therapy that lacks the risks of MSCs. As expected, subcutaneous BMSC-EV treatment significantly improved BLM-induced dermal damage and abnormalities and reduced ECM deposition and collagen density. The therapeutic effects of the BMSC-EVs were as strong as those of the BMSCs. Therefore, BMSC-EVs have great potential to be developed as a new therapy for SSc.

EVs serve as carriers that transport functional proteins, mRNAs, and miRNAs to various cells, where these factors act as mediators of intercellular communication and signaling pathways [42, 43]. Studies have proven that MSC-EV miRNAs possess the abilities to promote cell proliferation, accelerate injured tissue repair, and inhibit fibrotic diseases [16, 44–46]. In this study, we further demonstrated that there are a series of miRNAs in BMSC-EVs that contribute to the alleviation of SSc by regulating relevant signaling pathways. Previously, the TGFβ pathway, Toll-like receptor signaling, and the WNT pathway were reported to be the main dysfunctional signaling pathways in the skin of patients with SSc [47], and the TGFβ and Wnt/β-catenin pathways have been found to be hyperactivated to promote ECM production and induce fibrosis [48, 49]. In this study, we focused on these signaling pathways, and our data suggest that BMSC-EVs might treat BLM-induced SSc by regulating the TGFβ and WNT pathways as well as the inflammatory response. In a fibroblast SSc model induced by TGF-β1 modulation, the SSc markers COL1, FN1, and α-SMA and the inflammatory factors IL-6 and IL-10 were upregulated in the TGF-β1-treated fibroblast, and the increase in proteins was inhibited by the BMSC-EVs (Supplemental Fig. 2a). Additionally, the WNT signaling pathway proteins β-CATENIN and LEF1 and the TGFβ signaling pathway proteins phosphorylated Smad2 and phosphorylated Smad3 were upregulated in TGFβ1-treated fibroblasts and suppressed in BMSC-EV-treated fibroblasts (Supplemental Fig 2b). EVs inhibited fibroblast activation by downregulating the expression of TGF-β1 in the dermis, significantly reduced the infiltration of inflammatory cells and inhibited the release of inflammatory factors in the skin of BLM-induced SSc mice. All these effects were related to the miRNAs of the BMSC-EVs.

The miRNA let-7 family contains let-7a, 7b, 7c, 7d, 7e, 7f, 7 g, 7i, and 7j, mir-29, mir-125, and mir-21. All these miRNAs were found to be highly expressed in mouse BMSC-EVs in this study. let-7 was the first discovered miRNA and is functionally conserved in vertebrates [50]; in addition, it was reported to inhibit the production of proinflammatory cytokines such as Il8 and receptors such as Il1r1 and Il23r to negatively regulate the differentiation of Th17 cells and to regulate natural killer T cells [51–53]. Both EVs and miRNAs can regulate the TGFβ signaling pathway. For example, the downregulation of the Let-7, mir-29, and mir-30 families in idiopathic pulmonary fibrosis is related to the TGFβ pathway [54, 55], and mir-29 knockdown significantly upregulates TGFβ signaling in the induction of pulmonary fibrosis [54]. Moreover, let-7 cooperates with miR-99a and miR-125b, both of which are highly expressed in BMSC-EVs, when targeting receptor subunits and SMAD signaling transducers to block the TGFβ pathway [56]. TGFβ signaling and Wnt signaling have been found to promote each other to induce fibrosis in SSc [57, 58]. Mir-21 and mir-29 have been reported to target transducers of Wnt signaling [59, 60]. Thus, miRNAs from BMSC-EVs could regulate dysfunctional signals from pathways like the TGFβ and Wnt pathways to ameliorate SSc symptoms, including ECM deposition and inflammatory infiltration. This study increases the understanding of the molecular regulation of EV-mediated miRNAs in SSc pathogenesis and of the treatment of SSc with BMSC-EVs.

## Conclusion

BMSC-derived EVs could effectively treat the dysfunction and fibrosis of skin in a murine SSc model, demonstrating their potential as a replacement for related stem cell therapies. The miRNAs of BMSC-EVs might alleviate ECM deposition and inflammatory infiltration by regulating the TGFβ and WNT signaling pathways. For the first time, BMSC-EVs were proven to be able to intervened SSc in mice, and BMSC-EVs may provide a potential cure for patients with SSc.

## Supplementary Information

**Additional file 1: Supplemental Fig. 1.** GO analysis of the DEGs between BLM-induced SSc and control skin and between EV-treated and control skin. (a-b) GO Chord analysis results showing the Gene Ontology functions of differentially expressed genes between BLM-induced SSc skin and normal skin (a) and between BMSC-EV-treated skin and normal skin (b).

**Additional file 2: Supplemental Fig. 2.** BMSC-derived EVs suppress the TGF-β1-induced myofibroblast differentiation of mouse fibroblasts. (a) Protein expression profiles of SSc-related ECM and inflammatory factors in vitro. (b) Proteins related to WNT signaling pathway and TGFβ signaling pathway activation.

**Additional file 3.**

**Additional file 4.**

## Data Availability

RNA-seq data generated in the study can be accessed via the Gene Expression Omnibus under the accession codes GSE164965 and GSE165117. (https://www.ncbi.nlm.nih.gov/geo/query/acc.cgi?acc = GSE164965 and https://www.ncbi.nlm.nih.gov/geo/query/acc.cgi?acc = GSE165117). All other data are included within the article and its additional files.

## Abbreviations

BLM: Bleomycin
BMSC: Bone marrow mesenchymal stem cell
ECM: Extracellular matrix
EVs: Extracellular vesicles
FBS: Fetal bovine serum
GO: Gene Ontology
HRP: Horseradish peroxidase
IL: Interleukin
ROS: Reactive oxygen species
MSC: Mesenchymal stem cell
PBS: Phosphate-buffered saline
PFA: Paraformaldehyde
SSc: Systemic sclerosis
TEM: Transmission electron microscopy
TGFβ: Transforming growth factor β
