# Supplementary figures and images for: BMSC-derived extracellular vesicles intervened the pathogenic changes of scleroderma in mice through miRNAs
Source: Stem Cell Res Ther. 2021 Jun 5;12:327. doi: 10.1186/s13287-021-02400-y (PMC8179710; doi:10.1186/s13287-021-02400-y)

a

## BLM vs Normal

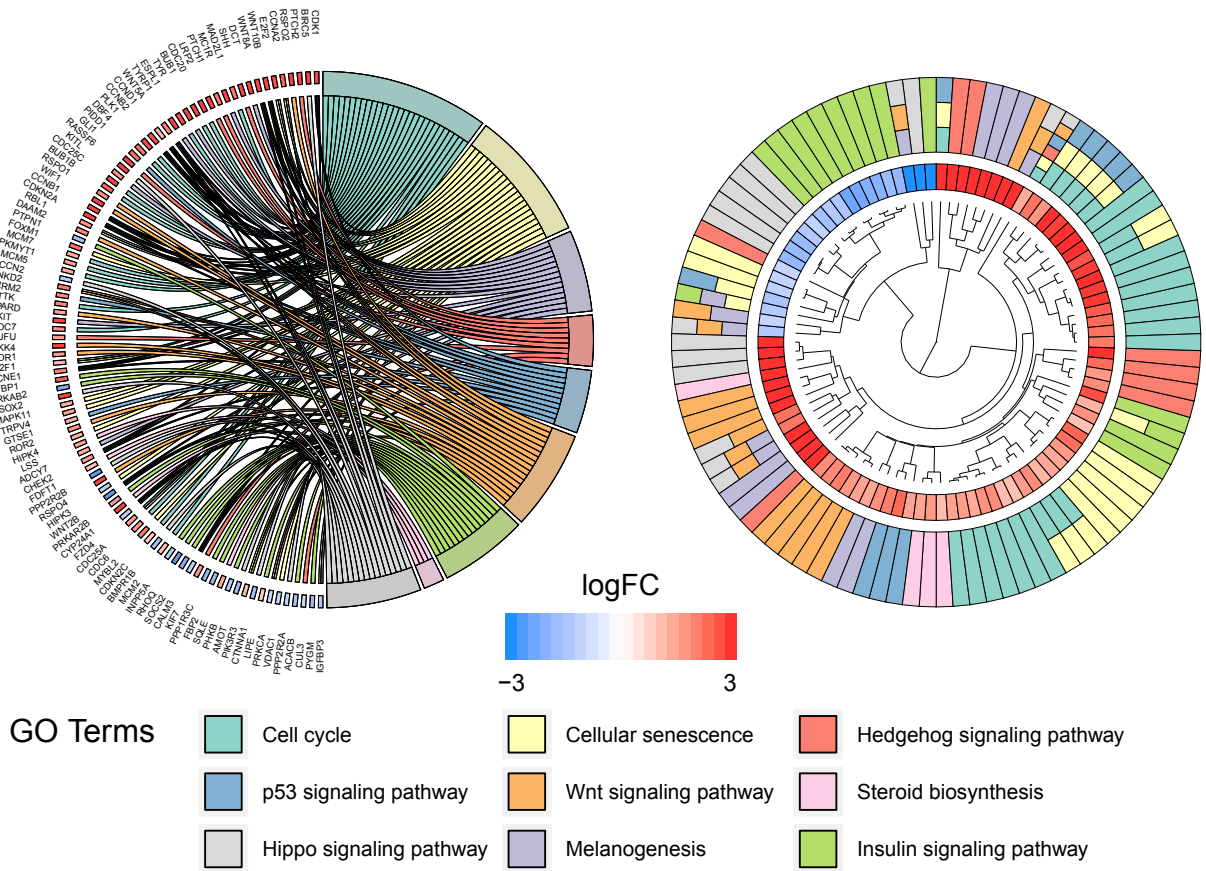

b

## EVs vs Normal

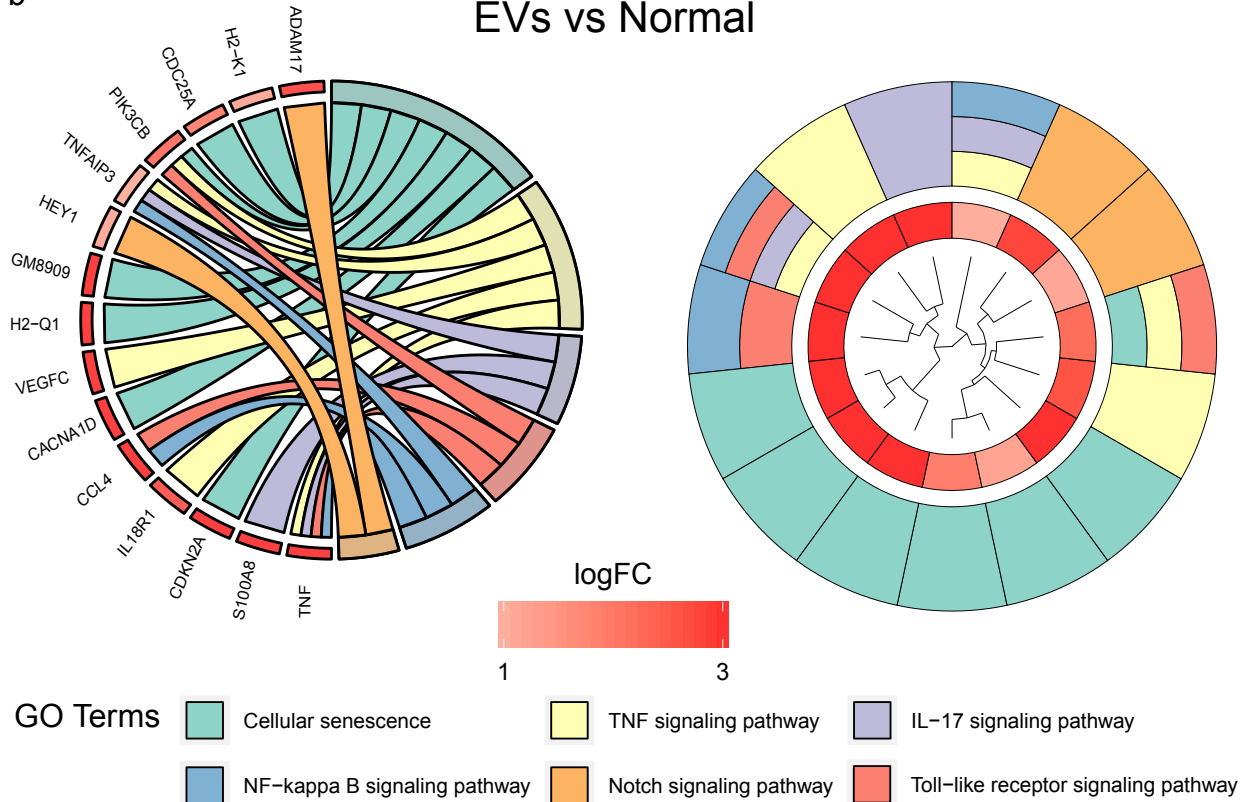

Supplement: Supplementary file 1 — Additional file 1: Supplemental Fig. 1. GO analysis of the DEGs between BLM-induced SSc and control skin and between EV-treated and control skin. (a-b) GO Chord analysis results showing the Gene Ontology functions of differentially expressed genes between BLM-induced SSc skin and normal skin (a) and between BMSC-EV-treated skin and normal skin (b). [file 13287_2021_2400_MOESM1_ESM.pdf]

**a**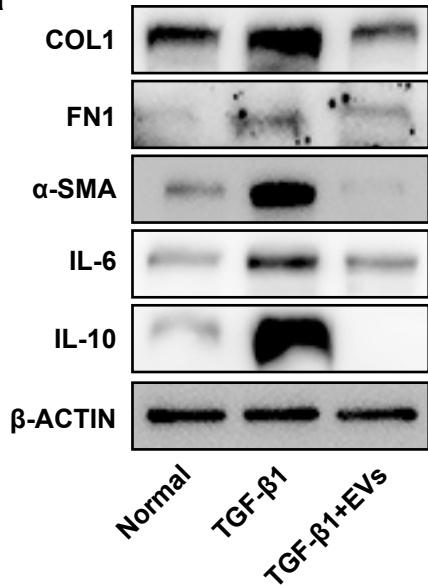**b**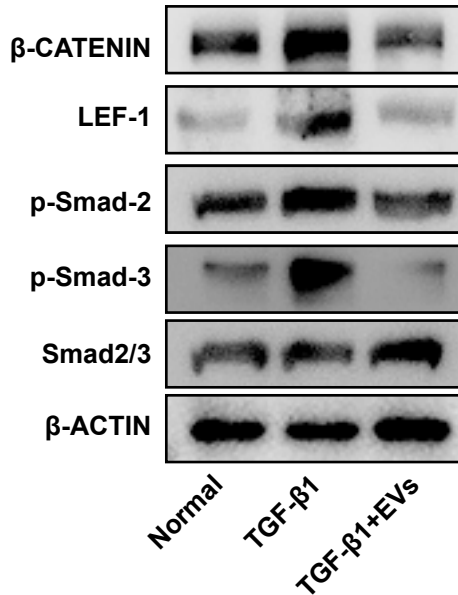

Supplement: Supplementary file 2 — Additional file 2: Supplemental Fig. 2. BMSC-derived EVs suppress the TGF-β1-induced myofibroblast differentiation of mouse fibroblasts. (a) Protein expression profiles of SSc-related ECM and inflammatory factors in vitro. (b) Proteins related to WNT signaling pathway and TGFβ signaling pathway activation. [file 13287_2021_2400_MOESM2_ESM.pdf]
