# Supplementary material for: BMSC-derived extracellular vesicles intervened the pathogenic changes of scleroderma in mice through miRNAs
Source: Stem Cell Res Ther. 2021 Jun 5;12:327. doi: 10.1186/s13287-021-02400-y (PMC8179710; doi:10.1186/s13287-021-02400-y)
Supplement: Supplementary file 3 — Additional file 3. [file 13287_2021_2400_MOESM3_ESM.docx]

**Supplemental Table 1: Antibodies used in immunostaining, Flow Cytometry and WB**

| Antibodies | Cat No. | Brand | Dilution ratio |
| --- | --- | --- | --- |
| CD9 | Ab92726 | Abcam | 1:1000 |
| CD63 | Ab217345 | Abcam | 1:1000 |
| Alix  Calnexin | 12422-1-AP  10427-2-AP | Proteintech  Proteintech | 1:1000  1:1000 |
| TGF-β1 | Ab92486 | Abcam | 1:100 |
| α-Sma | #25229 | Cell Signaling Technology | 1:100 |
| CD8 | #98941 | Cell Signaling Technology | 1:200 |
| CD4 | #25229 | Cell Signaling Technology | 1:50 |
| F4/80 | #30325S | Cell Signaling Technology | 1:50 |
| GAPDH | 60004-1-IG | Proteintech | 1:5000 |
| HRP-Conjugated Beta Actin Monoclonal Antibody | HRP-6008 | Proteintech | 1:10000 |
| Fn1 | 15613-1-AP | Proteintech | 1:1000 |
| Il-6 | 12912 | Cell Signaling Technology | 1:1000 |
| Il-10 | 20850-1-AP | Proteintech | 1:1000 |
| Tnf-α | 60291-1-ig | Proteintech | 1:1000 |
| β-Catenin | #8480 | Cell Signaling Technology | 1:1000 |
| Lef-1 | #2230 | Cell Signaling Technology | 1:1000 |
| Phospho-SMAD2 (Ser465/Ser467) | #18338 | Cell Signaling Technology | 1:1000 |
| Phospho-Smad3 (Ser423/425) | #9520 | Cell Signaling Technology | 1:1000 |
| Col1 | 14695-1-AP | Proteintech | 1:1000 |
| Hes1 | 11988S | Cell Signaling Technology | 1:200 |
| CD44 | 560596 | BD Pharmingen | 1:100 |
| CD90 | 553013 | BD Pharmingen | 1:100 |
| CD73 | 12-0731-81 | BD Pharmingen | 1:100 |
| CD105 | 12-1051-81 | BD Pharmingen | 1:100 |
| Scal1 | 553079 | BD Pharmingen | 1:100 |
| CD45  CD31 | 103108  102405 | BioLegend  BioLegend | 1:100  1:100 |

**Supplemental Table 2:Primers used in RT-PCR experiments**

| Primer | Forward (5’-3’) | Reverse (5’-3’) |
| --- | --- | --- |
| Col1 | ACCTGTGTGTTCCCTACT | GGTCATGCTCTCTCCAAAC |
| α-Sma | CCCAAGGAAAGGTAGGTGATAG | GGTGATGATGCCGTGTTCTA |
| Fn1 | CCTGGTTTGTACCTGCTATG | TGTAAGTGTTCCCAGTGTATTT |
| Tgf-β1 | GGTGGTATACTGAGACACCTTG | CCCAAGGAAAGGTAGGTGATAG |
| Il6 | GAGGAGACTTCACAGAGGATA | ATTTCCACGATTTCCCAGAG |
| Il10 | TAACGGAAACAACTCCTTGG | CACAGAGAAGCTCAGTGAATAA |
| Tnf-α | GTCTACTCCCAGGTTCTCTT | GGTTGACTTTCTCCTGGTATG |
| Gapdh | ATGGGGAAGGTGAAGGTCG | GGGGTCATTGATGGCAATA |
| 18S | GGACCAGAGCGAAAGCATTTG | GCCAGTCGGCATCGTTTATG |
| β-Actin | GGCTGAGGACTTTGTACATT | GGACTTCCTGTAACCACTTATT |
